# Kakonein restores diabetes‐induced endothelial junction dysfunction via promoting autophagy‐mediated NLRP3 inflammasome degradation

**DOI:** 10.1111/jcmm.16747

**Published:** 2021-06-27

**Authors:** Dawei Lian, Jiaying Liu, Ruifang Han, Jiaqi Jin, Li Zhu, Yanhong Zhang, Yi Huang, Xiao Wang, Shaoxiang Xian, Yang Chen

**Affiliations:** ^1^ The First Affiliated Hospital and Postdoctoral Research Station Guangzhou University of Chinese Medicine Guangzhou China; ^2^ School of Pharmaceutical Guangzhou University of Chinese Medicine Guangzhou China; ^3^ Laboratory Animal Center Guangzhou University of Chinese Medicine Guangzhou China; ^4^ Department of Stomatology The School of Dental Medicine Jinan University First Affiliated Hospital Guangzhou China; ^5^ Department of Traditional Chinese Medicine School of Medicine Guangzhou First People’s Hospital South China University of Technology Guangzhou China

**Keywords:** autophagy, diabetes, endothelial dysfunction, kakonein, NLRP3 inflammasome

## Abstract

In diabetes‐induced complications, inflammatory‐mediated endothelial dysfunction is the core of disease progression. Evidence shows that kakonein, an isoflavone common in *Pueraria*, can effectively treat diabetes and its complications. Therefore, we explored whether kakonein protects cardiovascular endothelial function by inhibiting inflammatory responses. In this study, C57BL/6J mice were injected with streptozocin to establish a diabetes model and treated with kakonein or metformin for 7 days. The protective effect of kakonein on cardiovascular endothelial junctions and NLRP3 inflammasome activation was verified through immunofluorescence and ELISA assay. In addition, the regulation of autophagy on the NLRP3 inflammasome was investigated through Western blot, immunofluorescence and RT‐qPCR. Results showed that kakonein restored the function of endothelial junctions and inhibited the assembly and activation of the NLRP3 inflammasome. Interestingly, kakonein decreased the expression of NLRP3 inflammasome protein by not reducing the transcriptional levels of NLRP3 and caspase‐1. Kakonein activated autophagy in an AMPK‐dependent manner, which reduced the activation of the NLRP3 inflammasome. In addition, kakonein inhibited both hyperglycaemia‐induced cardiovascular endothelial junction dysfunction and NLRP3 inflammasome activation, similar to autophagy agonist. Our findings indicated that kakonein exerts a protective effect on hyperglycaemia‐induced chronic vascular disease by regulating the NLRP3 inflammasome through autophagy.

## 1 INTRODUCTION

The incidence rate of diabetes and its complications is high worldwide.^1^ A large number of studies have shown that elevated blood sugar can lead to excessive inflammation to damage the vascular endothelium^2^, ^3^ and cause complications, such as atherosclerotic plaque instability, rupture and even cardiovascular emergencies.^4^, ^5^, ^6^, ^7^ Therefore, inhibiting inflammation‐mediated endothelial dysfunction may be a new way to prevent complications related to hyperglycaemia.

Chinese herbal medicine has a long history in the prevention and control of diabetes; Puerariae Lobatae Radix (PLR) is an important prescription ingredient in the treatment of diabetes.^8^, ^9^ Kakonein, also known as puerarin, is major bioactive ingredient amongst the PLR isoflavones, and it has potential therapeutic effects on diabetes and its complications through anti‐inflammatory or antioxidant pathways.^10^ Although kakonein has been used to treat vascular diseases in China,^11^ its mechanism remains unclear to date. Many mechanisms affect vascular disorders, and our previous study reported that the NLRP3 inflammasome affects the occurrence and development of vascular diseases by regulating endothelial junction function.^12^, ^13^, ^14^ The NLRP3 inflammasome can aggregate and produce a variety of inflammatory mediators when stimulants are detected,^15^ and autophagy is considered an effective regulator of NLRP3 inflammasome activation in hyperglycaemia‐associated vascular complications.^16^, ^17^ Hence, the mechanism of kakonein in hyperglycaemia‐induced endothelial dysfunction through NLRP3 inflammasome needs to be further explored.

In this study, an experiment was designed to investigate the protective effect of kakonein and its potential mechanism. Results proved that kakonein weakened endothelial connectivity by inhibiting the activation of the NLRP3 inflammasome, which is significantly dependent on autophagic NLRP3 protein degradation.

## 2 MATERIALS AND METHODS

### 2.1 Animal procedures

C57BL/6J (8 weeks old, weighing 20‐24 g, male) were used in all experiments. All protocols were approved by the Institutional Animal Care and Use Committee of Guangzhou University of Chinese Medicine (Guangzhou, China). Mice were intraperitoneally injected with 100 mg/kg streptozocin (STZ; Sigma‐Aldrich), and fasting blood glucose levels were monitored every other day. Mice were considered diabetic when their fasting glucose levels were above 10.5 mmol L^−1^ for three consecutive days. The treatment group was subjected to intragastric administration with kakonein (20, 40 or 80 mg/kg, daily) or metformin (200 mg/kg, daily) for 7 days.

### 2.2 Cell culture and treatment

Mouse vascular endothelial cells (MVECs) were purchased from ATCC. The cells were maintained in DMEM containing 10% foetal bovine serum and 1% penicillin‐streptomycin (Gibco) in incubators. The MVECs were treated with kakonein (25‐75 μM) or metformin (2 mM) in response to control (glucose, 5.5 mM) or high glucose (HG; glucose, 30 mM) for 24 h. Meanwhile, the cells were treated with MCC950 (10 nM), rapamycin (10 nM) or 3‐MA (1 mM) as inhibitors or activator.

### 2.3 Confocal immunofluorescence microscopy

The cardiovascular tissue and cell samples were stained using rabbit rabbit‐ZO‐1 (1:200, Invitrogen, #RA231621,), rabbit rabbit‐ZO‐2 (1:200, Invitrogen, #QG218845), goat anti‐NLRP3 (1:200; Abcam, #ab4207,), mouse anti‐HMGB1 (1:200; Santa, #sc‐135809,), mouse anti‐Caspase‐1 (1:200; Santa, #sc‐56036), rabbit anti‐ASC (1:100, Santa, #sc‐22514‐R), mouse anti‐P62 (1:200; Abcam, #ab56416), mouse anti‐vWF (1:500; Abcam, #ab11713). After incubation with primary antibodies, the samples were washed and labelled with the corresponding Alexa Fluor‐488 (1:100, Invitrogen, #1853312) and Alexa Fluor‐555 (1:100, Invitrogen, #1843680) conjugated secondary antibodies. Fluorescence was visualized with a Zeiss LSM800 microscope. Co‐localization was analysed by Image‐Pro Plus software, and the co‐localization coefficient was calculated using Pearson's correlation coefficient as previously described.

### 2.4 ELISA assay

After treatment, the cell supernatant and mice serum were collected; then IL‐1β (R&D System, #MLB00C) and HMGB1 (R&D System, #MAB16901) production were measured by ELISA according to the protocol described by the manufacturer.

### 2.5 Western blot

Total protein was extracted using RIPA buffer (Thermo). The supernatant was centrifuged after 10 000 g for 15 min at 4℃, and protein concentration was measured with a BCA Protein Assay Kit (Beyotime). Cell homogenates were denatured with reducing Laemmli SDS‐sample buffer and boiled in a metal bath for 5 min at 95℃. Equal amounts of the protein samples were 40 μg and separated by 12% SDS‐PAGE and transferred onto a PVDF membrane. The membrane was incubated with primary antibodies at 4℃ overnight and then treated with anti‐rabbit IgG (1:1500; CST, #5127) or anti‐mouse IgG (1:1500; CST, #93702) for 2 h at room temperature. The primary antibodies were anti‐NLRP3 (1:1000; Abcam, ab91413#), anti‐Caspase‐1 (1:1000; Santa, #sc‐56036), anti‐LC3 (1:1500; CST, #Q9H492), anti‐P62 (1:2000; Abcam, #ab56416), anti‐AMPK (1:1500; CST, #5832) and anti‐P‐AMPK (1:1000; CST, #50081). Anti‐β‐actin (1:1000; BOSTER, #BM0627) was used as an internal control. The target bands were detected and analysed using ImageJ software (NIH).

### 2.6 Real‐time PCR analysis

Total RNA was extracted by TRIZOL reagent (Invitrogen) and reversed transcribed into cDNA and PCR‐amplified using a one‐step RT‐PCR kit with SYBR Green (Takara, JPN). Real‐time quantitative PCR was performed using a real‐time PCR system (CFX96, Applied Biosystems). The primers were synthesized as follows: 5′‐AGGAGAATGGACCTGCAAGC‐3′ (forward primer) and 5′‐TCTACCATCATCCAGCCTTGG‐3′ (reverse primer) for the mouse *Nlrp3* gene; 5′‐ GGCGAGAGAGGTGAACAAGG‐3′ (forward primer) and 5′‐ GCCAAGGTCTCCAGGAACAC‐3′ (reverse primer) for the mouse *Caspase‐1* gene; and 5′‐CCCATCTATGAGGGTTACGC‐3′ (forward primer) and 5′‐TTTAATGTCACGCACGATTTC‐3′ (reverse primer) for *β‐actin* (used as an internal reference control). The results were quantified using the 2^‐ΔΔCT^ method.

### 2.7 Statistical analysis

All data were analysed by SPSS 20.0 (Dunnett's test) and expressed as mean ±SEM. Statistical significance was set at *P* < .05 or *P* < .01.

## 3 RESULT

### 3.1 Kakonein restores endothelial tight junction proteins in the coronary arteries of hyperglycaemic mice

**FIGURE 1:**
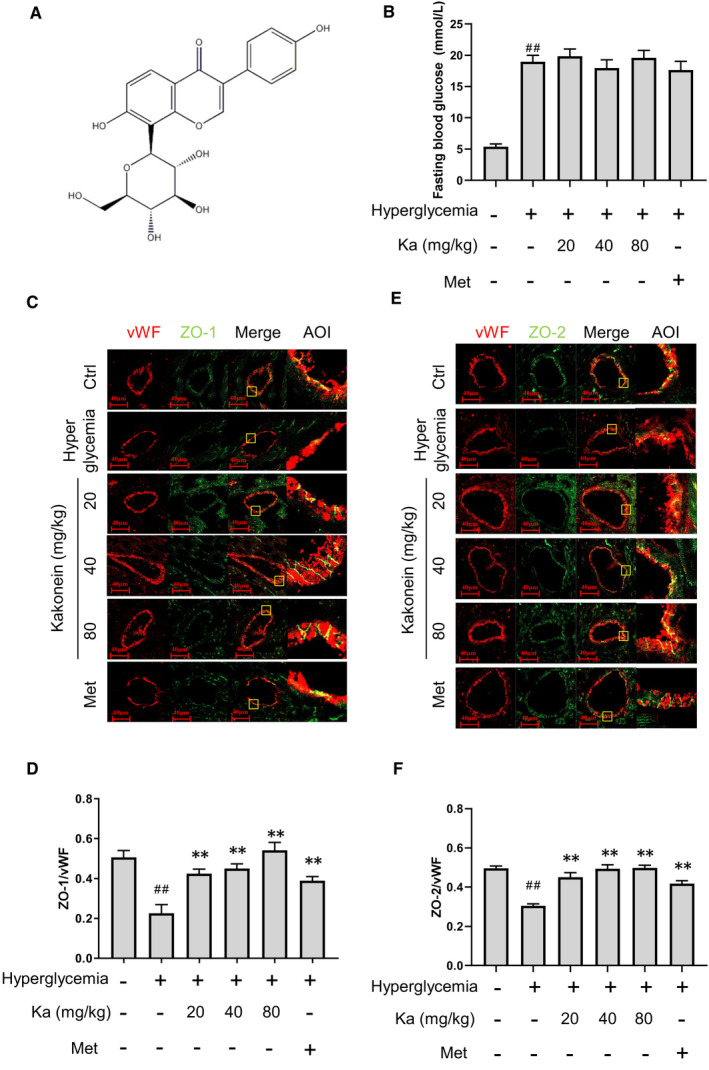
Therapeutic effects of kakonein on cardiac inter‐endothelial junction disruption in hyperglycaemic mice. (A) Molecular structural formula of puerarin. (B) Effect of kakonein on fasting blood glucose in hyperglycaemic mice (n = 8). (C) Fluorescence indicating the effect of kakonein or metformin hydrochloride on ZO‐1 (green) with VWF (red) co‐localization. (D) Quantitative analysis of the co‐localization of ZO‐1 with vWF (n = 8). (E) Fluorescence indicating the effect of kakonein or metformin hydrochloride on ZO‐2 (green) with VWF (red) co‐localization. (F) Quantitative analysis of the co‐localization of ZO‐2 with vWF (n = 8). ^##^ *P* < .01 compared with the control. **P < .01 compared with hyperglycaemia

Destruction of endothelial junctions occurs in the early stages of diabetic cardiovascular complications. In our experiments, the hyperglycaemic mice model was established, and oral therapy with kakonein (20, 40 and 80 mg/kg) or metformin (200 mg/kg) was administered for 7 days. After the intervention, we found that the fasting blood glucose of hyperglycaemic mice did not decrease (Figure 1B), but the expression of tight junction proteins ZO‐1 and ZO‐2 evidently increased under treatment with kakonein or metformin (Figure 1C and 1D). Thus, kakonein could repair endodermal permeability, not through the hypoglycaemic effect, in vivo.

### 3.2 Kakonein inhibits endothelial NLRP3 inflammasome activation in coronary arteries of hyperglycaemic mice

**FIGURE 2:**
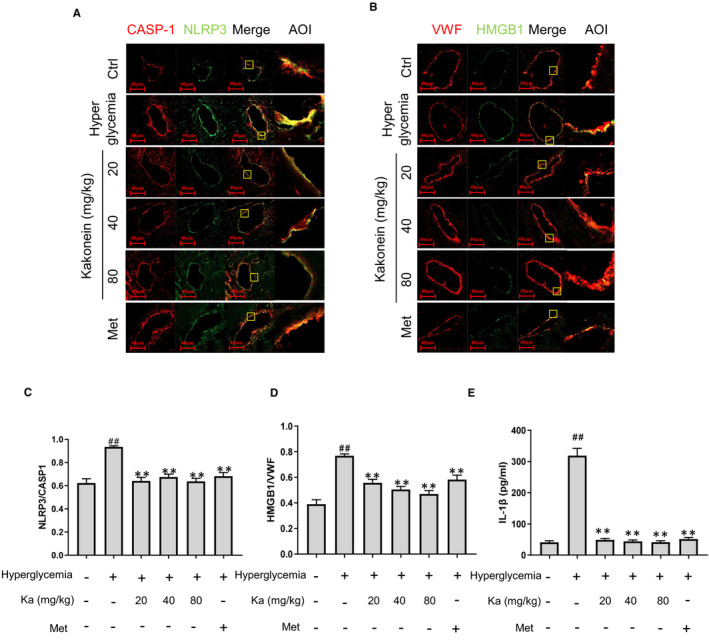
Kakonein inhibits NLRP3 inflammasome activation of cardiac vascular endothelium in hyperglycaemic mice. (A) Fluorescence indicating the effect of kakonein or metformin hydrochloride on NLRP3 (green) with caspase‐1 (red) co‐localization in cardiac endothelial cells. (B) Quantitative analysis of the co‐localization of NLRP3 with caspase‐1 (n = 8). (C) Fluorescence indicating the effect of kakonein or metformin hydrochloride on HMGB1 (green) with VWF (red) co‐localization. (D) Quantitative analysis of the co‐localization of HMGB1 with vWF (n = 8). (E) IL‐1β content in serum was detected by ELISA kit (n = 8). ^##^ *P* < .01 compared with the control. **P < .01 compared with hyperglycaemia

Our previous studies proved that the NLRP3 inflammasome mediated by hyperglycaemia is the core mechanism of endothelial dysfunction. Therefore, we explored whether the recovery of endothelial junction function by kakonein is related to the NLRP3 inflammasome. Through immunofluorescence observation, we found that kakonein inhibited the formation of the NLRP3 inflammasome (Figure 2A and 2B) and reduced the expression levels of HMGB1 (Figure 2C and 2D) and IL‐1β (Figure 2E) in hyperglycaemic mice.

### 3.3 Kakonein repairs the integrity of the endothelium by inhibiting the NLRP3 inflammasome

**FIGURE 3:**
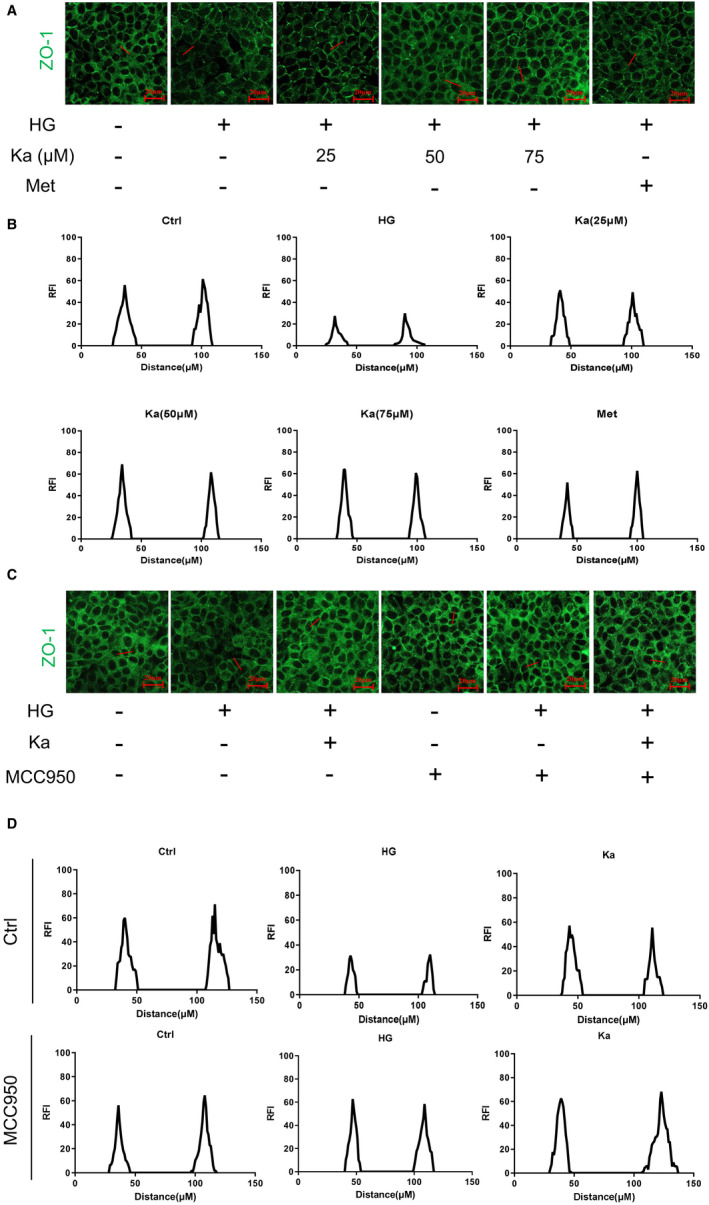
Therapeutic effect of kakonein on the recovery of the integrity of the endothelium under high glucose by inhibiting the NLRP3 inflammasome. (A) Representative fluorescence images of ZO‐1 (n = 4). (B) ZO‐1 expression was represented by a histogram of fluorescence intensity (RFI). (C) Representative images of immunofluorescence indicating the effects of MCC950 and kakonein on ZO‐1 under high‐glucose stimulation (n = 4). (D) ZO‐1 expression was represented by a histogram of fluorescence intensity (RFI)

To verify that the therapeutic effect of kakonein is related to the inhibition of NLRP3 inflammasome activation, we established a model of endothelial junction destruction induced by HG and used MCC950 (a selective NLRP3 inflammasome inhibitor) for treatment. As shown in Figure 3A and 3B, MVECs stimulated with HG decreased ZO‐1 expression, which was obviously restored by kakonein or metformin. However, in the presence of MCC950, the therapeutic effect disappeared (Figure 3C and 3D). These data indicated that inhibiting NLRP3 inflammasome activation was a key mechanism for kakonein to restore endothelial integrity.

### 3.4 Kakonein inhibits endothelial NLRP3 inflammasome assembly and activation in endothelial cells

**FIGURE 4:**
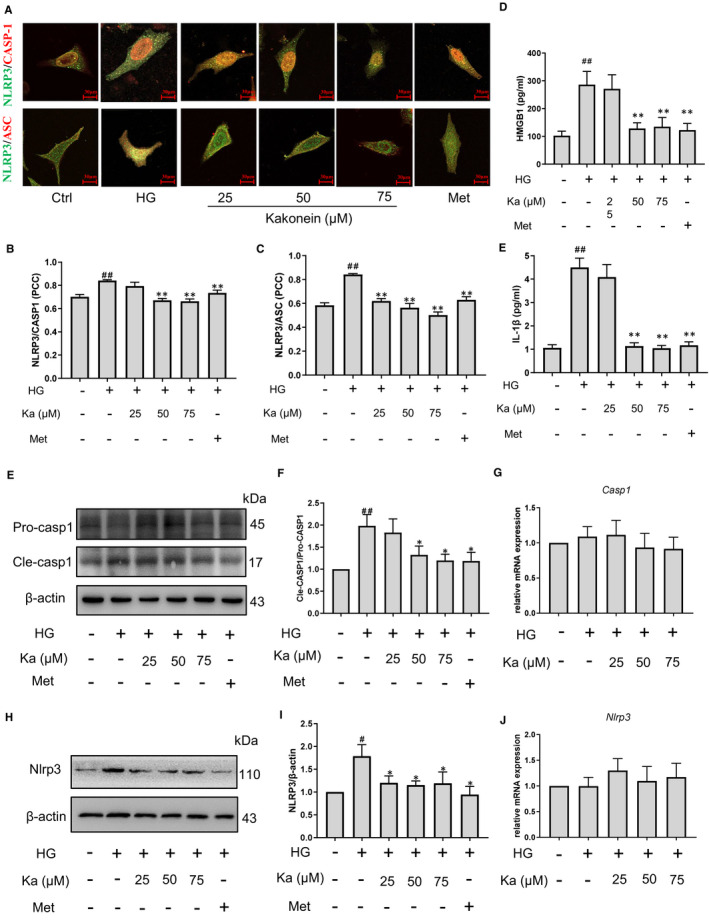
Effect of kakonein on high glucose‐induced NLRP3 inflammasome assembly and activation in endothelial cells. (A) Fluorescence indicating the effect of kakonein and metformin hydrochloride on NLRP3 (green) with caspase‐1 (first line, red) or ASC (second line, red) co‐localization. (B) and (C) Quantitative analysis of the co‐localization of NLRP3 with caspase‐1 or ASC (n = 4). (D) IL‐1β content in supernatant was detected by ELISA kit (n = 4). (E) and (F) Analysis and summary of the effect of kakonein and metformin hydrochloride on caspase‐1 expression through Western blot (n = 4). (G) Analysis of *Caspase‐1* transcriptional level (n = 4). (H) and (I) Analysis and summary of the effect of kakonein and metformin hydrochloride on NLRP3 expression through Western blot (n = 4). (J) Analysis of *Nlrp3* transcriptional level (n = 4). ^##^ *P* < .01 compared with the control. **P < .01 compared with HG. *P < .05 compared with HG

Immunofluorescence was used to detect the assembly of the NLRP3 inflammasome in MVECs. Results showed that co‐localization of NLRP3 and caspase‐1 decreased in the kakonein groups compared with the model group. Similar results were detected in Figure 4A–4C, which demonstrated that kakonein could inhibit the aggregation and formation of NLRP3 inflammasomes. The protein expression of NLRP3 and caspase‐1 and its downstream product IL‐1β and HMGB1 significantly decreased in the HG model with kakonein treatment (Figure 4D–4I). However, kakonein suppressed NLRP3 inflammasome protein expression by not reducing the transcription levels of *Nlrp3* and *Caspase‐1* (Figure 4G–4J). These results indicated that kakonein may reduce activated NLRP3 inflammasome through post‐translational protein modification.

### 3.5 Kakonein restores endothelial autophagy in hyperglycaemia

**FIGURE 5:**
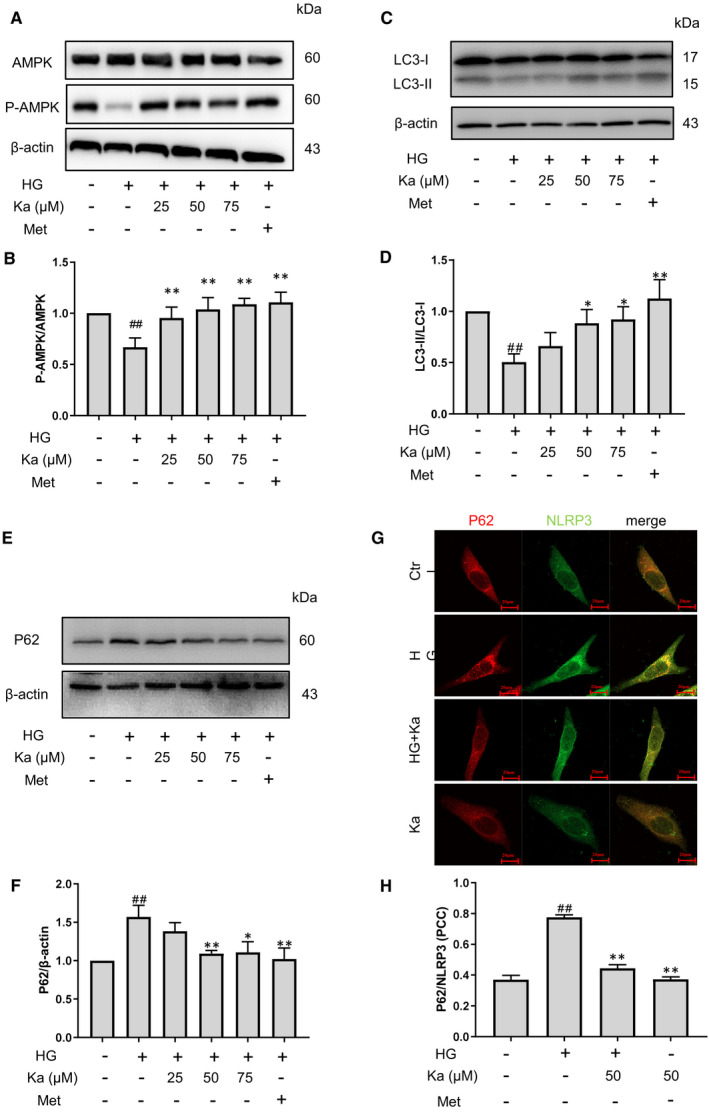
Recovery of high glucose‐induced decrease of autophagy in endothelial cells by kakonein (A) and (B) Analysis and summary of high glucose‐induced AMPK expression through Western blot (n = 4). (C) and (D) Analysis and summary of high glucose‐induced LC3 expression through Western blot (n = 4). (E) and (F) Analysis and summary of high glucose‐induced P62 expression through Western blot (n = 4). (G) Fluorescence indicating the effect of kakonein on NLRP3 (green) and P62 (red) co‐localization in endothelial cells. (H) Quantitative analysis of the co‐localization of NLRP3 with P62 (n = 4). ^#^ *P* < .05 and ^##^ *P* < .01 compared with the control. *P < .05 and **P < .01 kakonein or metformin hydrochloride compared with HG

In hyperglycaemia, the inhibition of AMPK‐dependent autophagy is an important factor leading to inflammation. As shown in Figure 5A and 5B, we found that kakonein could restore the AMPK pathway in HG, which suggested that kakonein may activate autophagy through the AMPK pathway. We investigated the autophagy state and found that kakonein treatment significantly elevated LC3‐II expression and reduced P62 expression (Figure 5C–5F). Meanwhile, HG stimulation increased the co‐localization of P62 and NLRP3 in endothelial cells, whereas kakonein intervention reduced the co‐localization of P62 and NLRP3 (Figure 5G and 5H). Therefore, kakonein could restore the autophagy pathway to degrade the NLRP3 inflammasome in HG.

### 3.6 Recovery of autophagy activity abolishes hyperglycaemia‐induced dysfunction of endothelial junctions

**FIGURE 6:**
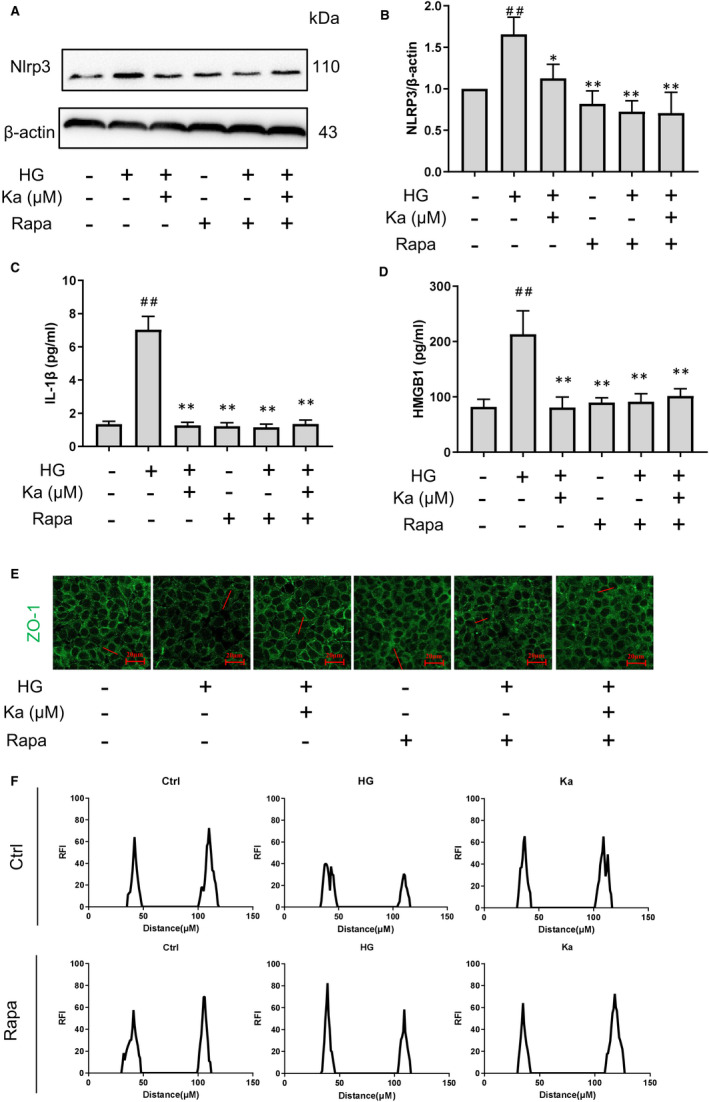
Validation of the therapeutic effect of kakonein in HG‐induced endothelial cell dysfunction through the upregulation of autophagy. (A) and (B) Analysis and summary of NLRP3 expression after rapamycin intervention through Western blot (n = 4). (C) IL‐1β content in supernatant was detected by ELISA kit (n = 4). (D) HMBG1 content in supernatant was detected by ELISA kit (n = 4). (E) Immunofluorescence representative images indicating the effects of rapamycin and kakonein on ZO‐1 under high‐glucose stimulation (n = 4). (F) The expression of ZO‐1 was represented by RFI. ^#^ *P* < .05 compared with the control. **P < .01 and *P < .05 compared with HG

We found that rapamycin, an autophagy agonist, reduced NLRP3 protein expression (Figure 6A and 6B) and the NLRP3 inflammasome product of HMGB1 (Figure 6C) and IL‐1β (Figure 6D) and then restored HG‐induced endothelial junction disruption (Figure 6E and 6F). Meanwhile, the therapeutic effects of kakonein disappeared in the presence of rapamycin. By contrast, 3‐MA, an autophagy inhibitor, increased NLRP3 protein expression and abolished the effects of kakonein (Figure S7). Thus, these results indicated that the therapeutic effect of kakonein was through the recovery of autophagy to inhibit the NLRP3 inflammasome.

## 4 DISCUSSION

Previous studies have shown that the NLRP3 inflammasome is related to the destruction of endothelial cell connections caused by hyperglycaemia, leading to hyperglycaemic complications.^7^ Simultaneously, an increasing number of studies have found that Chinese herbal medicines and its extracts have obvious therapeutic effects on vascular endothelial dysfunction induced by diabetes.^18^ Kakonein, an important PLR isoflavone, is also used in the clinical adjuvant therapy of cardiovascular disease^19^ and shows obvious anti‐inflammatory effect.^20^ Our results demonstrated that kakonein protects against hyperglycaemia*‐*induced endothelial dysfunction by restoring endothelial autophagy to degrade the NLRP3 inflammasome.

Previous studies have demonstrated that kakonein has anti‐diabetic effects by protecting pancreatic β‐cells from STZ damage.^21^ Thus, in our study, kakonein was administered only after STZ destroyed the pancreatic β‐cells of mice to prevent kakonein from protecting blood vessels through the insulin pathway. The results showed that kakonein did not affect blood glucose (Figure 1B) and ameliorated the damage of the junction of cardiac vascular endothelium caused by hyperglycaemia (Figure 1C–1F). Although it has been reported that kakonein's hypoglycaemic effect,^22^ we further found that kakonein could significantly reduce the fasting blood glucose (Figure S1) and ameliorated the junction of cardiac vascular endothelium (Figure S2) for 4 weeks in the dose range of 80 mg/kg. After eliminating the hypoglycaemic effect, a model of endothelial junctions in an HG environment was established. Without obvious cytotoxic effects (Figure S3), kakonein restored endothelial junction function after HG stimulation in vitro (Figure 3). Metformin, which exhibits anti‐inflammatory and pro‐autophagic effects for the treatment of hyperglycaemic complications,^23^, ^24^, ^25^ served as a positive control drug and achieved the same effect as kakonein in protecting endothelial junction function in hyperglycaemia. Although metformin has been shown to exert anti‐inflammatory effects, kakonein shows its potential advantages in other aspects. For example, kakonein could lower the levels of urinary albumin excretion, serum creatinine (CRE) and blood urea nitrogen (BUN), meanwhile upregulated the heparan sulphate proteoglycan expression and creatinine clearance rate diabetic nephropathy.^26^ The same result was found that kakonein restored kidney damage in hyperglycaemic animals at 4 weeks. In our supplementary experiment, however, metformin did not (Figure S4). Furthermore, kakonein not only dose‐dependently suppressed HG‐induced vasoconstriction and vasodilation dysfunction, but also augmented myocardium metabolism and amelioration of the cardiac function.^20^ These data revealed that kakonein played a key role in the recovery of hyperglycaemia‐induced endothelial junction dysfunction independent of reducing blood glucose.

As an important intracellular signalling platform in the endothelium, inflammasomes play an important role in the innate immune response, including regulating endothelial junction function in hyperglycaemia.^7^ Although immune cells are more likely to activate inflammasomes, our experimental data showed that there was no macrophage infiltration in cardiovascular tissue (Figure S5). Therefore, we focussed on the inflammasome of vascular endothelial cells. Recently, obvious anti‐inflammatory effects have been found in numerous Chinese medicines for the treatment of diabetes and its complications, including *Pueraria lobata* (Wild.) Ohwi.^27^, ^28^, ^29^ Our findings showed that kakonein decreased the protein expression levels of NLRP3 and caspase‐1 but did not change the gene transcription levels of NLRP3 and caspase‐1 (Figures 2 and 4). Therefore, kakonein could reduce the activation of the NLRP3 inflammasome through the degradation pathway.

Intracellular protein degradation mainly includes the ubiquitin pathway, autophagy pathway and caspase pathway, amongst which autophagy dysfunction is considered an important pathogenic mechanism of diabetes.^30^, ^31^ Recent research proved that the increase of ROS in high glucose suppressed the binding between LKB1 and AMPKα, which reduces p‐AMPKα T172 levels, resulting in AMPK inactivation.^32^ It is well known that AMPK, a key enzyme in maintaining metabolic homeostasis, is closely related to the decline of autophagy in diabetes.^33^ Meanwhile, induction of autophagy leading to inactivation or selective clearance of the NLRP3 inflammasome regulates vascular disease in hyperglycaemia.^34^, ^35^, ^36^ This study determined whether kakonein attenuates the activation of the NLRP3 inflammasome through the autophagy pathway. The results showed that kakonein could increase LKB1‐AMPKα binding which suppressed by high glucose (Figure S6) and restore AMPK activity, result to enhance autophagy under HG conditions (Figure 5A–5F). Meanwhile, autophagosomes containing abundant NLRP3 accumulated under HG stimulation, and kakonein could significantly reduce the accumulation of autophagosomes (Figure 5G and 5H).

To complement this anti‐inflammatory mechanism, we treated endothelial cells with rapamycin, which acted as an autophagy agonist, as another positive control drug. Similar to kakonein, rapamycin inhibited HG‐induced NLRP3 inflammasome activation and protected the function of endothelial junctions (Figure 6). In addition, the autophagy inhibitor 3‐methyladenine significantly increased the expression of NLRP3, thereby confirming our hypothesis (Figure S7). Although rapamycin could significantly increase the level of autophagy in cells, its adverse effects are often reported, such as stomatitis and myositis^37^; non‐infectious interstitial pneumonitis and increasing the severity of bacterial infections^38^; anaemia and leukopenia.^39^ On the contrary, kakonein has been used in the clinical adjuvant treatment of cardiovascular‐related diseases in China.^40^ There are fewer reports of adverse reactions, and it is safer than rapamycin.

In conclusion, this study reported for the first time that kakonein has a significant cardiovascular protective effect, which is reflected in the restoration of endothelial junctions by inhibiting the NLRP3 inflammasome through activation of autophagy in hyperglycaemia. The results of this study may be helpful to guide the medication of kakonein as an anti‐inflammatory agent in patients with chronic metabolic inflammation. The progress of cardiovascular disease could be delayed with kakonein.

## CONFLICTS OF INTEREST

The authors have declared that no competing interest exists.

## AUTHOR CONTRIBUTION

**Dawei Lian:** Data curation (equal); Formal analysis (equal); Investigation (equal); Methodology (equal); Writing‐original draft (equal). **Jiaying Liu:** Data curation (equal); Formal analysis (equal); Investigation (equal); Writing‐original draft (equal). **Ruifang Han:** Data curation (equal); Formal analysis (equal); Investigation (equal); Writing‐original draft (equal). **Jiaqi Jin:** Investigation (supporting); Methodology (supporting). **Zhu Li:** Investigation (supporting); Methodology (supporting). **Yanhong Zhang:** Funding acquisition (supporting); Resources (supporting). **Yi Huang:** Methodology (supporting); Resources (supporting). **Xiao Wang:** Funding acquisition (equal); Project administration (equal); Resources (equal). **Shaoxiang Xian:** Funding acquisition (equal); Project administration (equal); Resources (equal); Writing‐review & editing (equal). **Yang Chen:** Funding acquisition (equal); Project administration (equal); Resources (equal); Writing‐review & editing (equal).

## Supporting information

Supplementary Material

## References

[1] Khunti K , Gomes MB , Pocock S , et al. Therapeutic inertia in the treatment of hyperglycaemia in patients with type 2 diabetes: A systematic review. Diabetes Obes Metab. 2018;20(2):427‐437. https://doi.org10.1111/dom.13088 28834075 10.1111/dom.13088 PMC5813232

[2] De Vriese AS , Verbeuren TJ , Van de Voorde J , Lameire NH , Vanhoutte PM . Endothelial dysfunction in diabetes. Br J Pharmacol. 2000;130(5):963‐974. https://doi.org10.1038/sj.bjp.0703393 10882379 10.1038/sj.bjp.0703393 PMC1572156

[3] Wan Z , Fan Y , Liu X , et al. NLRP3 inflammasome promotes diabetes‐induced endothelial inflammation and atherosclerosis. Diabetes, Metabolic Syndrome and Obesity : Targets and Therapy. 2019;12:1931‐1942. https://doi.org10.2147/DMSO.S222053 10.2147/DMSO.S222053 PMC6759984 31571967

[4] Zernecke A , Weber C . Chemokines in the vascular inflammatory response of atherosclerosis. Cardiovasc Res. 2010;86(2):192‐201. https://doi.org10.1093/cvr/cvp391 20007309 10.1093/cvr/cvp391

[5] Moriya J . Critical roles of inflammation in atherosclerosis. J Cardiol. 2019;73(1):22‐27. https://doi.org10.1016/j.jjcc.2018.05.010 29907363 10.1016/j.jjcc.2018.05.010

[6] Fava C , Montagnana M . Atherosclerosis is an inflammatory disease which lacks a common anti‐inflammatory therapy: How human genetics can help to this issue. A Narrative Review. Frontiers in Pharmacology. 2018;9: 10.3389/fphar.2018 PMC5808208 29467655

[7] Chen Y , Wang L , Pitzer AL , Li X , Li PL , Zhang Y . Contribution of redox‐dependent activation of endothelial Nlrp3 inflammasomes to hyperglycemia‐induced endothelial dysfunction. J Mol Med. 2016;94(12):1335‐1347. https://doi.org10.1007/s00109‐016‐1481‐5 27783111 10.1007/s00109-016-1481-5 PMC5512566

[8] Zhang Z , Lam TN , Zuo Z . Radix Puerariae: An overview of its chemistry, pharmacology, pharmacokinetics, and clinical use. J Clin Pharmacol. 2013;53(8):787‐811. https://doi.org10.1002/jcph.96 23677886 10.1002/jcph.96

[9] Chen Z , Yuan Y , Zou X , et al. Radix Puerariae and Fructus Crataegi mixture inhibits renal injury in type 2 diabetes via decreasing of AKT/PI3K. BMC Complement Altern Med. 2017;17(1): 10.1186/s12906-017-1945-3 PMC5591499 28886733

[10] Ma TW , Wen YJ , Song XP , et al. Puerarin inhibits the development of osteoarthritis through antiinflammatory and antimatrix‐degrading pathways in osteoarthritis‐induced rat model. Phytother Res. 2021;35(5):2579‐2593. 10.1002/ptr.6988 33350519

[11] Cheng M , Li X , Guo Z , et al. Puerarin accelerates re‐endothelialization in a carotid arterial injury model: impact on vasodilator concentration and vascular cell functions. J Cardiovasc Pharmacol. 2013;62(4):361‐368. https://doi.org10.1097/FJC.0b013e31829dd961 23792702 10.1097/FJC.0b013e31829dd961

[12] Lian D , Lai J , Wu Y , et al. Cathepsin B‐mediated NLRP3 inflammasome formation and activation in Angiotensin II ‐induced hypertensive mice: Role of Macrophage digestion dysfunction. Cellular Physiology and Biochemistry: International Journal of Experimental Cellular Physiology, Biochemistry, and Pharmacology. 2018;50(4):1585‐1600. https://doi.org10.1159/000494656 10.1159/000494656 30359991

[13] Chen Y , Li X , Boini KM , et al. Endothelial Nlrp3 inflammasome activation associated with lysosomal destabilization during coronary arteritis. Biochem Biophys Acta. 2015;1853(2):396‐408. https://doi.org10.1016/j.bbamcr.2014.11.012 25450976 10.1016/j.bbamcr.2014.11.012 PMC4289419

[14] Chen Y , Pitzer AL , Li X , Li PL , Wang L , Zhang Y . Instigation of endothelial Nlrp3 inflammasome by adipokine visfatin promotes inter‐endothelial junction disruption: role of HMGB1. J Cell Mol Med. 2015;19(12):2715‐2727. https://doi.org10.1111/jcmm.12657 26293846 10.1111/jcmm.12657 PMC4687695

[15] Swanson KV , Deng M , Ting JP . The NLRP3 inflammasome: molecular activation and regulation to therapeutics. Nat Rev Immunol. 2019;19(8):477‐489. https://doi.org10.1038/s41577‐019‐0165‐0 31036962 10.1038/s41577-019-0165-0 PMC7807242

[16] Cao ZR , Wang YH , Long ZM , He GQ . Interaction between autophagy and the NLRP3 inflammasome. Acta Bioch Bioph Sin. 2019;51(11):1087‐1095. https://doi.org10.1093/abbs/gmz098 10.1093/abbs/gmz098 31609412

[17] Zhong Z , Sanchez‐Lopez E , Karin M . Autophagy, NLRP3 inflammasome and auto‐inflammatory/immune diseases. Clin Exp Rheumatol. 2016;34(4 Suppl 98):12‐16 27586797

[18] Oduro PK , Fang J , Niu L , et al. Pharmacological management of vascular endothelial dysfunction in diabetes: TCM and western medicine compared based on biomarkers and biochemical parameters. Pharmacol Res. 2020;158: https://doi.org10.1016/j.phrs.2020.104893 10.1016/j.phrs.2020.104893 32434053

[19] Ulbricht C , Costa D , Dam C , et al. An evidence‐based systematic review of kudzu (Pueraria lobata) by the Natural Standard Research Collaboration. Journal of Dietary Supplements. 2015;12(1):36‐104. https://doi.org10.3109/19390211.2014.904123 24848872 10.3109/19390211.2014.904123

[20] Zhou YX , Zhang H , Peng C . Puerarin: a review of pharmacological effects. Phytother Res. 2014;28(7):961‐975. https://doi.org10.1002/ptr.5083 24339367 10.1002/ptr.5083

[21] Yang L , Yao D , Yang H , et al. Puerarin Protects Pancreatic beta‐Cells in Obese Diabetic Mice via Activation of GLP‐1R Signaling. Mol Endocrinol. 2016;30(3):361‐371. https://doi.org10.1210/me.2015‐1213 26789107 10.1210/me.2015-1213 PMC5414651

[22] Li Z , Shangguan Z , Liu Y , et al. Puerarin protects pancreatic β‐cell survival via PI3K/Akt signaling pathway. J Mol Endocrinol. 2014;53(1):71‐79. https://doi.org10.1530/jme‐13‐0302 24827001 10.1530/JME-13-0302

[23] Bailey CJ . Metformin: effects on micro and macrovascular complications in type 2 diabetes. Cardiovasc Drugs Ther. 2008;22(3):215‐224. https://doi.org10.1007/s10557‐008‐6092‐0 18288595 10.1007/s10557-008-6092-0

[24] Shi WY , Xiao D , Wang L , et al. Therapeutic metformin/AMPK activation blocked lymphoma cell growth via inhibition of mTOR pathway and induction of autophagy. Cell Death Dis. 2012;3(3):e275. https://doi.org10.1038/cddis.2012.13 22378068 10.1038/cddis.2012.13 PMC3317343

[25] Xie ZL , Lau K , Eby B , et al. Improvement of cardiac functions by chronic metformin treatment is associated with enhanced cardiac autophagy in diabetic OVE26 mice. Diabetes. 2011;60(6):1770‐1778. https://doi.org10.2337/db10‐0351 21562078 10.2337/db10-0351 PMC3114402

[26] Liu CM , Ma JQ , Sun YZ . Puerarin protects rat kidney from lead‐induced apoptosis by modulating the PI3K/Akt/eNOS pathway. Toxicol Appl Pharmacol. 2012;258(3):330‐342. https://doi.org10.1016/j.taap.2011.11.015 22172631 10.1016/j.taap.2011.11.015

[27] Huang WY , Wang HJ , Alzhan A , Tian WX , Ma XF . Traditional Chinese medicines with anti‐inflammatory functions and their inhibitory effects on fatty acid synthase. Prog Biochem Biophys. 2020;47(8):809‐817. https://doi.org10.16476/j.pibb.2020.0140

[28] Zhou JX , Liu TH , Wu LL . Regulation of Chinese herbal medicines in inflammation of diabetic nephropathy. Prog Biochem Biophys. 2020;47(8):818‐834. https://doi.org10.16476/j.pibb.2020.0188

[29] Lertpatipanpong P , Janpaijit S , Park EY , Kim CT , Baek SJ . Potential anti‐diabetic activity of Pueraria lobata flower (Flos Puerariae) extracts. Molecules. 2020;25(17):3970‐ 10.3390/molecules25173970 PMC7504745 32878147

[30] Kim J , Lim YM , Lee MS . The role of autophagy in systemic metabolism and human‐type diabetes. Mol Cells. 2018;41(1):11‐17. https://doi.org10.14348/molcells.2018.2228 29370692 10.14348/molcells.2018.2228 PMC5792707

[31] Levine B , Kroemer G . Autophagy in the pathogenesis of disease. Cell. 2008;132(1):27‐42. https://doi.org10.1016/j.cell.2007.12.018 18191218 10.1016/j.cell.2007.12.018 PMC2696814

[32] Jiang P , Ren L , Zhi L , et al. Negative regulation of AMPK signaling by high glucose via E3 ubiquitin ligase MG53. Mol Cell. 2021;81(3):629‐637.e5. https://doi.org10.1016/j.molcel.2020.12.008 33400924 10.1016/j.molcel.2020.12.008

[33] Madhavi YV , Gaikwad N , Yerra VG , Kalvala AK , Nanduri S , Kumar A . Targeting AMPK in diabetes and diabetic complications: energy homeostasis, autophagy and mitochondrial health. Curr Med Chem. 2019;26(27):5207‐5229. https://doi.org10.2174/0929867325666180406120051 29623826 10.2174/0929867325666180406120051

[34] Yu SX , Du CT , Chen W , et al. Genipin inhibits NLRP3 and NLRC4 inflammasome activation via autophagy suppression. Sci Rep. 2016;5(1):1‐12. https://doi.org10.1038/srep17935 10.1038/srep17935 PMC4675967 26659006

[35] Cao Z , Wang Y , Long Z , He G . Interaction between autophagy and the NLRP3 inflammasome. Acta Biochim Biophys Sin (Shanghai). 2019;51(11):1087‐1095. https://doi.org10.1093/abbs/gmz098 31609412 10.1093/abbs/gmz098

[36] Hou Y , Lin S , Qiu J , et al. NLRP3 inflammasome negatively regulates podocyte autophagy in diabetic nephropathy. Biochem Biophys Res Comm. 2020;521(3):791‐798. https://doi.org10.1016/j.bbrc.2019.10.194 31703838 10.1016/j.bbrc.2019.10.194

[37] Weiner SM , Sellin L , Vonend O , et al. Pneumonitis associated with sirolimus: clinical characteristics, risk factors and outcome–a single‐centre experience and review of the literature. Nephrology, dialysis, transplantation : official publication of the European Dialysis and Transplant Association ‐ European Renal Association. 2007;22(12):3631‐3637. https://doi.org10.1093/ndt/gfm420 10.1093/ndt/gfm420 17611248

[38] Dunn JLM , Kartchner LB , Gast K , et al. Mammalian target of rapamycin regulates a hyperresponsive state in pulmonary neutrophils late after burn injury. J Leukoc Biol. 2018;103(5):909‐918. https://doi.org10.1002/jlb.3ab0616‐251rrr 29393976 10.1002/JLB.3AB0616-251RRR PMC6181446

[39] Ceschi A , Heistermann E , Gros S , et al. Acute sirolimus overdose: a multicenter case series. PLoS One. 2015;10(5):e0128033. https://doi.org10.1371/journal.pone.0128033 26020944 10.1371/journal.pone.0128033 PMC4447358

[40] Cai Y , Zhang J , He Y , et al. A Supramolecular Hydrogel of Puerarin. J Biomed Nanotechnol. 2018;14(2):257‐266. https://doi.org10.1166/jbn.2018.2483 31352922 10.1166/jbn.2018.2483

