# Supplementary material for: Kakonein restores diabetes‐induced endothelial junction dysfunction via promoting autophagy‐mediated NLRP3 inflammasome degradation
Source: J Cell Mol Med. 2021 Jun 27;25(15):7169–80. doi: 10.1111/jcmm.16747 (PMC8335672; doi:10.1111/jcmm.16747)
Supplement: Supplementary file 1 — Supplementary Material [file JCMM-25-7169-s001.docx]

**Supplementary Materials:**

1. **Blood glucose test**

The fasting blood glucose of the mice was observed from 1 week to 4 weeks after the establishment of hyperglycemia model. The blood glucose in the model group was significantly higher than that of other groups and kakonein group decreased significantly in the third and fourth week, while metformin group did not change significantly (Figure S1).


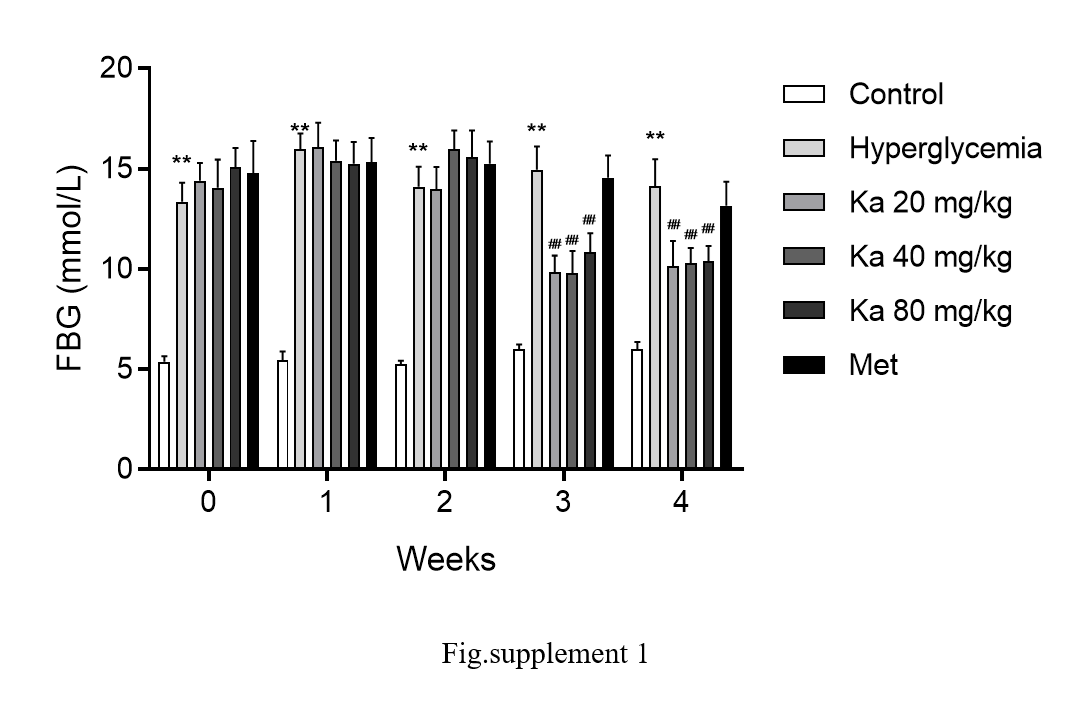


(A) Blood glucose was observed for four weeks after establishment of hyperglycemia model in each group (n=8).

1. **Detection of endothelial junction function**

Immunofluorescence was used to detect the endothelial junction function. Results showed that the endothelial connection proteins ZO-1 and ZO-2 were significantly recovered in the kakonein group in fourth week (Figure S2).


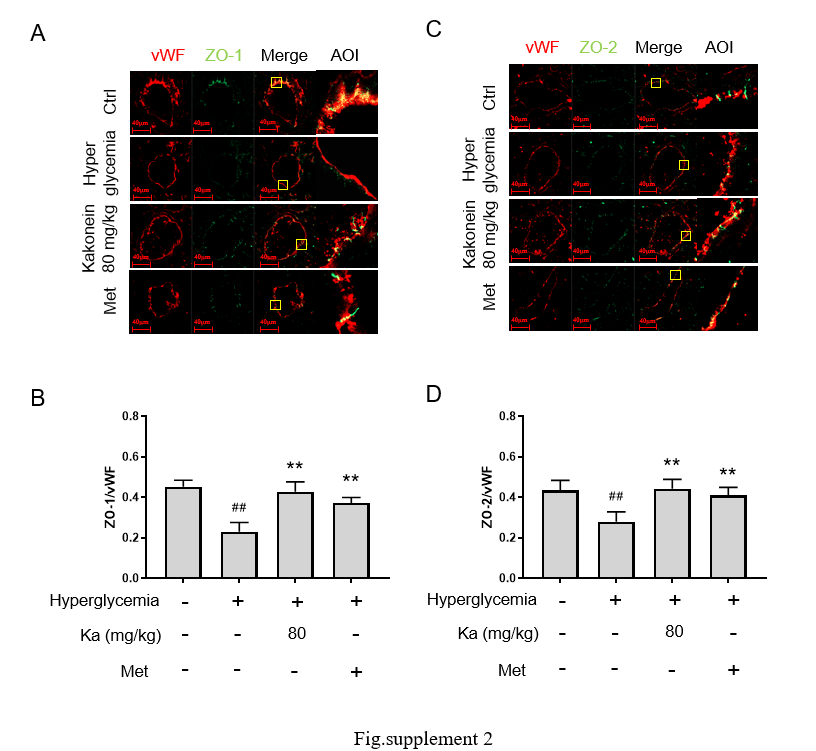


(A) Fluorescence indicating the effect of kakonein on ZO-1 (green) with VWF (red) co-localisation. (B) Quantitative analysis of the co-localisation of ZO-1 with vWF (n=6). (C) Fluorescence indicating the effect of kakonein on ZO-2 (green) with VWF (red) co-localisation. (D) Quantitative analysis of the co-localisation of ZO-2 with vWF (n=6).

1. **Cell viability**

We determined cell viability after administration of different concentrations of kakonein for intervention; There was no change in cell viability at concentration was between 12.5-100 (μM) (Figure S3 A). Then, after administration of different concentrations of kakonein or metformin under the stimulation of high glucose, there was no significant change in cell viability (Figure S3 B).


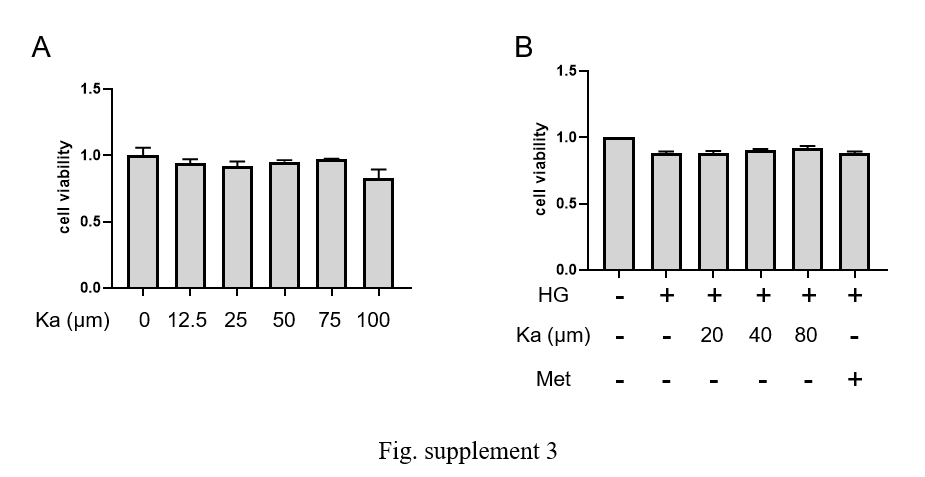


(A) Cell viability after administration of different concentrations of kakonein intervention (n=6). (B) Cell viability after administration of different concentrations of kakonein and metformin interventions (n=6).

1. **Renal function test**

The serum creatinine (CRE) and blood urea nitrogen (BUN) of the mice was observed from 1 week to 4 weeks after the establishment of hyperglycemia model. The results showed that serum CRE and BUN did not change significantly in hyperglycemic animals in one week, however both indicators significant increased in the fourth week. Kakonein could significantly reduce the level of CRE and BUN, but metformin had no similar effect (Figure S4).


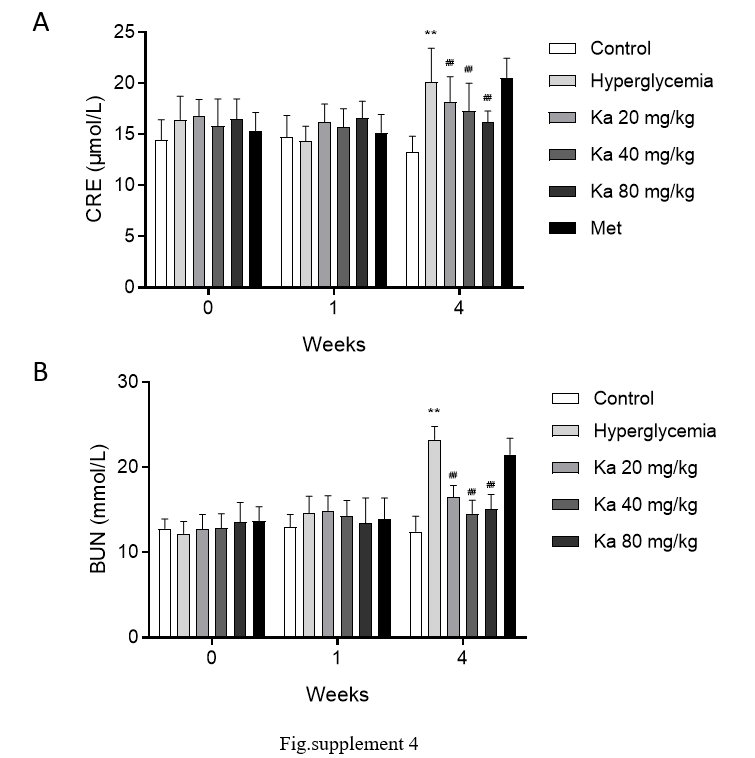


(A) The serum creatinine was observed for four weeks after establishment of hyperglycemia model in each group (n=8). (B) The blood urea nitrogen was observed for four weeks after establishment of hyperglycemia model in each group (n=8).

1. **Inflammatory infiltration test**

Immunofluorescence was used to detect the infiltration of immune inflammatory cells in cardiovascular tissue. Results showed that there was no macrophage infiltration in cardiovascular tissue (Figure S5).


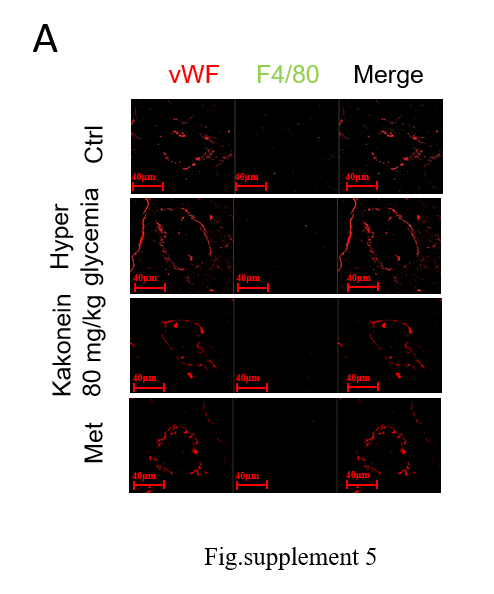


(A) Fluorescence indicating the effect of kakonein on F4/80 (green) with VWF (red) co-localisation (n=6).

1. **Immunofluorescence test**

Immunofluorescence was used to detect the assembly of the LKB1 and AMPKα in MVECs. Results showed that co-localisation of LKB1 and AMPKα decreased in the HG groups compared with the control group, while kakonein could restore the co-localization of LKB1 and AMPKα. (Figure S6).


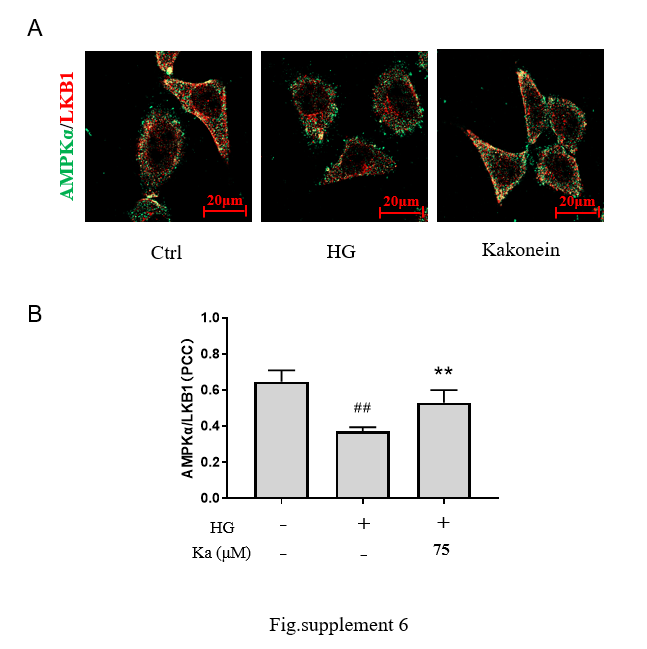


(A) Fluorescence indicating the effect of kakonein on AMPKα (green) with LKB1(red) co-localisation. (B) Quantitative analysis of the co-localisation of AMPKα and LKB1 (n=4).

1. **Effect of a****utophagy inhibitor 3-methyladenine on NLRP3 expression**

MVECs were given 3-MA intervention, cells were extracted and used western blotting to analyze the NLRP3 protein expression. The result showed that NLRP3 protein expression in the cells was significantly increased in the 3-MA group. (Figure S7).


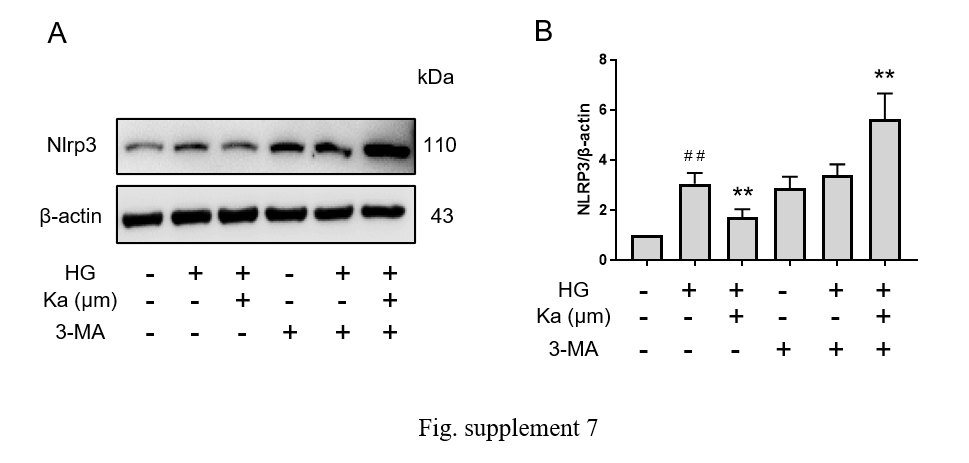


(A) and (B) Analysis and summarized showing the effect of autophagy inhibitor 3-methyladenine on NLRP3 expression though western blot (n=4). ^##^p < 0.01 compared with the control. **p < 0.01 and *p < 0.05 compared with the HG.
